# Exploring the mechanisms driving elderly Fintech engagement: the role of social influence and the elderly’s digital literacy

**DOI:** 10.3389/fpsyg.2024.1420147

**Published:** 2024-06-21

**Authors:** Yi Mei

**Affiliations:** School of Finance, Zhejiang University of Finance & Economics, Hangzhou, Zhejiang, China

**Keywords:** silver generation, digital literacy, social influence, elderly digital engagement, m-banking

## Abstract

**Objective:**

This study aims to investigate the elderly digital engagement (acceptance and utilization of technology), with a focus on the widespread application of financial technology: mobile banking (m-banking).

**Methods:**

Guided by social influence theory, the research examines the various social dynamics that encourage elderly engagement with m-banking and the moderating effects of their digital literacy. Data was gathered online utilizing a disjunctive approach and analyzed using Partial Least Squares Structural Equation Modeling (PLS-SEM).

**Results:**

The study reveals that both word-of-mouths (WOMs) and peer engagement significantly influence the elderly’s perceived usefulness of the platform, thereby influencing their m-banking engagement. Additionally, the level of digital literacy among older adults was found to impact their perceived usefulness of m-banking services. Interestingly, digital literacy among older adults negatively moderates the positive associations of WOMs and peer engagement on perceived usefulness.

**Discussion:**

These insights advance our understanding of how social interactions can steer technological engagement, particularly for the silver generation with diverse levels of digital literacy. As society ages and becomes increasingly digitized, it is imperative to promote digital engagement among the elderly and foster a more inclusive digital environment.

## 1 Introduction

Technological advancements have significantly altered financial service models, introducing considerable convenience into daily activities (Bongomin et al., 2024). M-banking, for instance, facilitates straightforward daily transactions and fund transfers via mobile devices, streamlining financial operations (Neves et al., 2024). Yet, the adoption of m-banking is not universal, with a lower rate observed among silver generation (adults over 50; Choudrie et al., 2020; Shareef et al., 2023). Despite the positive impact of new technologies on elderly well-being, challenges persist in their widespread acceptance and use (Wong et al., 2022). As financial technology progresses, it is vital to develop strategies for the elderly to engage with and benefit from these Fintech innovations, preventing their exclusion from the digital era.

Within the realm of technology adoption, older adults often require external motivation to recognize and utilize the benefits of new technologies (Vaportzis et al., 2017). This support can come from family recommendations or observed use by peers. Social influences are crucial as they affect how older adults think about and feel toward technology, influencing their digital engagement (Zhang S Y, et al., 2023). Despite the clear importance of these social aspects, research on leveraging this influence to promote elderly Fintech engagement is limited. Current studies mainly examine how technology features (Alvarez-Dardet et al., 2020), personal traits (Cajamarca and Herskovic, 2022; Begde et al., 2024), and motivational factors (Kadylak and Cotten, 2020; Bongomin et al., 2024) affect digital engagement, but the discussion on the social factors influencing the elderly’s digital engagement, particularly financial technology, is not sufficiently in-depth. With banks widely using social strategies to boost mobile banking (i.e., WOMs marketing and showcasing peer engagement; Wong et al., 2022; Sharma, 2024), and older adults heavily influenced by social factors in tech adoption, there is a clear need for profound research on social mechanisms to enhance elderly Fintech engagement.

Investigating mechanisms that enhance elderly engagement with financial technology necessitates accounting for their varied digital literacy. Elaboration-Likelihood Model (ELM; Zhang et al., 2024) theorizes how social influences affect this population based on their digital literacy. ELM distinguishes between central and peripheral information processing routes (Jeyaraj et al., 2023), with the former demanding a high level of cognitive engagement and critical thinking skills and the latter relying on trusted sources, such as family members, friends, or respected community groups, to inform decisions. Given the distinct cognitive demands and capabilities required by the central and peripheral routes, individuals with lower digital literacy are constrained by their abilities and are more prone to employ the peripheral route for analyzing matters related to financial technology. Existing research indicates that the digital literacy levels of the elderly vary (Moravec et al., 2024; Sakariassen and Ytre-Arne, 2024), there is a gap in the research regarding how these individual differences specifically influence the elderly’s receptiveness to social influences and their Fintech engagement.

This study therefore poses the following research questions:

*Research Question 1*: How is the elderly’s Fintech engagement (using m-banking as an example) influenced by social interactions?

*Research Question 2*: How do differences in digital literacy among older adults affect the extent to which they are socially influenced to engage in fintech?

The study applies social influence theory to thoroughly investigate the multifaceted social mechanisms by which elderly users on platforms are encouraged to adopt financial technology. Utilizing survey data, the research employs Partial Least Squares Structural Equation Modeling (PLS-SEM) to examine how the elderly’s social interactions (WOMs, peer engagement, and subjective norms) influence the elderly’s perceived usefulness of the platform, thereby affecting their m-banking engagement. Additionally, the study explores the role of the elderly’s digital literacy within this social influence pathway.

This research holds significant potential contributions as it offers a nuanced understanding of the factors that encourage the engagement with m-banking among the elderly, a critical aspect of financial technology. By identifying specific social influence mechanisms, the study can inform the development of more effective social interventions aimed at the elderly, addressing their unique needs. Furthermore, by emphasizing the importance of digital literacy, the research highlights the necessity for targeted initiatives that consider various levels of digital literacy among the elderly population, which is essential for their successful integration into the digital financial eco-system. This study could lead to the development of strategies that promote financial inclusion for the elderly, ensuring that they can fully participate in and benefit from the digital economy.

## 2 Literature review

### 2.1 The elderly in m-banking literature

The banking industry has witnessed a transformative technological shift in the form of m-banking, a service that leverages mobile devices such as cell phones, smartphones, and tablets to deliver financial products and services (Yang et al., 2023). This novel approach empowers customers to conduct a broad range of financial transactions and related activities using their mobile devices, including, among others, bill payments, fund transfers, and account balance checks (Bongomin et al., 2024). Moreover, the benefits of m-banking to the end-user are manifold, with a particular focus on increased convenience, the assurance of immediate and real-time access to various banking services, and the effective management of time (Ciunova-Shuleska et al., 2022). However, despite the widespread adoption of smartphones worldwide, financial institutions are still confronted with challenges stemming from consumers’ reluctance and skepticism toward the uptake and utilization of m-banking services, especially for certain segments of the population that are less technologically inclined, like the elderly (Wong et al., 2022). Considering the possible constraints on the cognitive processing abilities of the elderly, they may struggle to independently recognize the benefits and perceived utility of the emerging technology (i.e., mobile banking services) in the absence of external guidance, such as social influence (Paimre et al., 2023; Wilson-Nash et al., 2023).

The past decade has witnessed a marked escalation in the volume of scholarly investigations of factors driving the adoption of m-banking services (Neves et al., 2024; Shareef et al., 2024). However, the literature on m-banking appears to have neglected the silver generation, who represent an emerging and potentially lucrative market segment for financial institution (Bongomin et al., 2024). This group, typically are defined as individuals who are 50 years of age and older (Silva et al., 2022; Lin et al., 2024; Zhou et al., 2024), exhibits a lower propensity to adopt m-banking technologies when juxtaposed with their younger counterparts (Flick et al., 2020; Begde et al., 2024). Furthermore, the aging process is often accompanied by a decline in sensory and cognitive faculties, which can influence the manner in which older adults engage with and interact with novel technological advancements (Begde et al., 2024).

The existing body of research on the adoption and utilization of m-banking has repeatedly highlighted the need for investigations that concentrate on the elderly population. Wong et al. (2022) advocate for an increased focus on qualitative research endeavors aimed at uncovering the underlying factors that influence older adults’ decisions to adopt and consistently use financial services via smartphones. Echoing this sentiment, Sharma (2024) emphasize that academic research in the field of m-banking has often overlooked the elderly, stressing the importance of examining the multifaceted and intricate characteristics of this demographic in forthcoming studies. Given China’s aging demographic and supportive policies, studying the use of mobile banking by seniors over 50 is essential for understanding their economic actions, enhancing financial inclusion, and boosting societal welfare, which has profound theoretical and practical impacts on socio-economic development (Neves et al., 2024). By focusing on this age group, the study seeks to address the research gap and contribute to a more nuanced understanding of the experiences, challenges, and opportunities associated with m-banking engagement among older adults.

### 2.2 Elderly digital divide: intergenerational and intragroup perspectives

Empirical studies have demonstrated the presence of a digital divide not only within the elderly demographic but also in comparison to other age cohorts (Friemel, 2016; Zhao et al., 2023). Research indicates that younger generations are often considered digital natives of the current era, possessing higher levels of digital literacy and the ability to rapidly embrace and apply technological advancements (Bongomin et al., 2024). In contrast, the process of engagement with digital technology by the elderly is generally slower and more complex (Choudrie et al., 2018). While a segment of the elderly population actively adopts technology and readily uses information services, the majority, as digital immigrants, face challenges in technology adoption and usage due to a lack of extensive exposure during their formative years (Friemel, 2016; Wen et al., 2020). Numerous scholarly studies demonstrate a significant digital divide within the elderly population, and these disparities are difficult to overcome in a short period of time (Friemel, 2016). The digital divide among the elderly is not only reflected in their familiarity with technology but also encompasses various aspects including their propensity to seek out and embrace information technology, the regularity with which they engage with such technology, and their capacity to effectively utilize it for various purposes (Zhang Y H, et al., 2023). Since these differences are deeply rooted in a multitude of factors including individual cognitive abilities, educational background, economic status, and level of social participation, they are unlikely to disappear rapidly (Shang et al., 2022; Cammisuli et al., 2023; Wolf et al., 2023; Sharma, 2024).

Therefore, researching how to guide elderly individuals with varying levels of digital literacy to successfully integrate into digital society and fully leverage digital technology has become an important topic (Friemel, 2016). Existing literature indicates that, constrained by their limited digital literacy, the majority of elderly individuals rarely take the initiative to explore and grasp the appeal of technology (Pantelaki et al., 2023). Consequently, they often rely on the support and assistance from family, friends, and broader social networks to successfully integrate into the digital society (Wong et al., 2022). Consequently, developing social strategies to help the elderly overcome barriers to using digital technology and enhance their quality of life and social participation is crucial. To promote digital inclusion and engagement among the elderly, various measures can be taken, including but not limited to the establishment of supportive social networks and targeted social influence. Through such social impact measures, the digital divide can be effectively bridged, allowing the elderly to enjoy the conveniences and benefits brought by digital technology.

### 2.3 Social influence on the elderly in literature on technology

Social influence is crucial for technology adoption among the elderly, who often face specific challenges in digital inclusion (Maceviciute et al., 2023). Many seniors are hesitant to explore tech independently, highlighting the need for supportive networks to facilitate their digital interactions (Racin et al., 2023). The concept of “guanxi”(Li M Y, et al., 2023), or relationships, in social contexts is particularly powerful in fostering technology adoption among the elderly, particularly beneficial for those feeling isolated or unsure about technology (Li M Y, et al., 2023; Tang et al., 2023).

Drawing on literature concerning social influence on the elderly technology (Creech, 2019; Gong et al., 2022) and social influence theory (Trenz et al., 2018), we can summarize three social processes that guide the elderly to embrace technology (Table 1). One of the key social processes is internalization which integrates societal norms and digital tech values into their actions through exposure to the positive WOMs that often comes from elder’s trusted sources (Zhang et al., 2023). This WOM can highlight the specific advantages that technology offers, which may assist in unlocking the technological barriers for the elderly, especially those with limited digital literacy, allowing them to perceive the utility of technology and potentially embrace it. Another important social process is identification by emulating the portrayal of peers successfully using technology. Observing their peers using it can help dispel the elderly’s doubts and hesitations about the usefulness of the technology, motivating them to learn and explore it (Hopp et al., 2020), and fosters a sense of community and belonging by showing that they are part of a group that values technology (Colosimo and Badia, 2021). In cultures that emphasize the importance of strong social connections, the social norm to remain connected via technological platforms can act as a potent driver for engagement (Au, 2023). This facilitates older adults in preserving their social ties and fulfilling communal expectations, thereby exemplifying the social value that the technology imparts to this demographic (Ma et al., 2023).

**Table 1:** Social influence process on elderly digital engagement.

Social influence process	Individual goals	Triggers	Reference
Internalization	Acquire real information	WOMs	Choudrie et al. (2020), Ma et al. (2023), Shou et al. (2023)
Identification	Similar to peers	Peer Engagement	Wong et al. (2022), Zhang et al. (2023), Bongomin et al. (2024)
Compliance	Earn positive feedback from others	Subjective norms	Al-Muwil et al. (2019), Alvarez-Dardet et al. (2020)

## 3 Theoretical framework and hypothesis development

### 3.1 Theoretical framework

This study constructs a theoretical framework social influence theory (Trenz et al., 2018) to elucidate the mechanisms behind the adoption of m-banking technology by the elderly under social influence. Elderly individuals’ perceived usefulness of m-banking are regarded as the psychological mechanisms of their engagement with m-banking services. The judgment of perceived usefulness is influenced by social influence (Almogren and Aljammaz, 2022; Sajid et al., 2022).

According to social influence theory, the adoption of m-banking services among the elderly is significantly steered by social factors that operate through three social processes: internalization (positive WOM from trusted sources eases concerns and promotes tech exploration; Zhang et al., 2023), identification (peer engagement in technology reduces misunderstandings and enhances a sense of community; Colosimo and Badia, 2021), and compliance (social networks set tech-using norms, aiding in social connection and meeting expectations; Ma et al., 2023). These mechanisms directly impact the perceived usefulness of the technology, which is a critical determinant of adoption. WOMs from existing users of m-banking services acts as a pivotal element in the internalization process and lead to more favorable perceptions of the technology’s utility (Lim et al., 2024). The process of identification comes into play as elderly individuals observe and emulate the behaviors of their peers within their social circles (Jia et al., 2024). The desire to belong and maintain positive social relationships can motivate older adults to adopt m-banking services if they see them as being widely used and accepted by their peers. Subjective norms, which are the perceived expectations from valued guanxi, drive the compliance aspect of the social influence process (Sánchez-García et al., 2022; Zhou et al., 2024). When elderly individuals feel that their social circle expects them to use m-banking, they are more likely to engage with the service to align with these expectations, thereby attributing a social value to its use.

The elderly’s digital literacy is examined as a moderating factor in the interplay between social interaction and perceived usefulness of m-banking services (Bongomin et al., 2024). Digital literacy is about the individual’s ability to engage with technology, and perceived usefulness is about the perceived benefits and applicability of the technology (Oba and Berger, 2023). Digital literacy can modulate the relationship between social interaction and perceived use. The greater the digital literacy of the elderly, the more adept they are at harnessing digital information beyond interpersonal channels to form a comprehensive understanding of m-banking’s use (Shi et al., 2023; Tang et al., 2023); hence, digital literacy moderates the relationship between perceived usefulness and social interaction.

**Figure 1:**
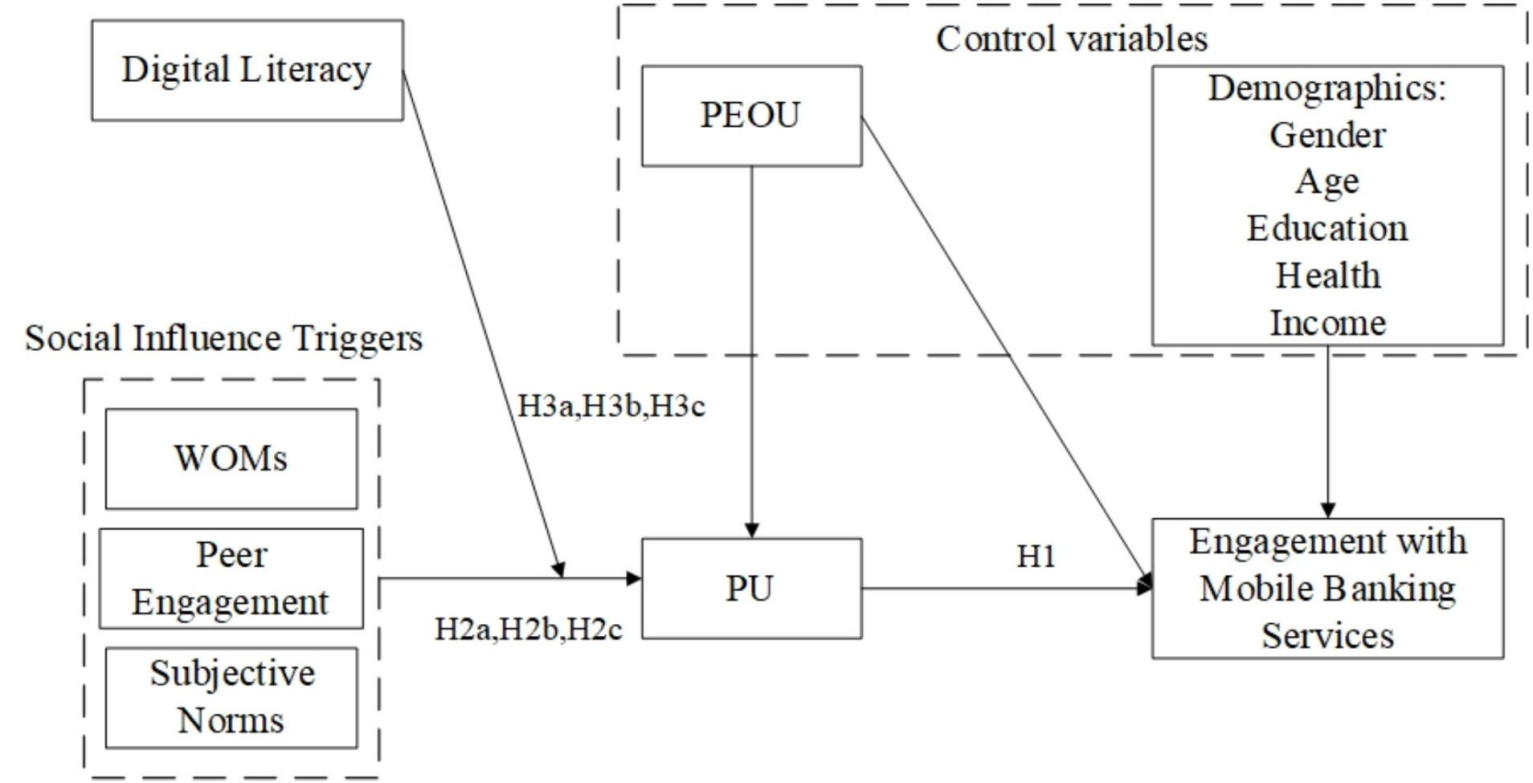
The proposed model (PU=Perceived usefulness; PEOU=Perceived ease of use).

Guided by Technology acceptance model (TAM; Davis, 1989) has been extensively utilized to predict and explain user acceptance of various technologies and applied in numerous studies across different contexts, including software, hardware, and services (Hu et al., 2024; Saif et al., 2024), perceived ease of use has been selected as a control variable affecting perceived usefulness and engagement. The impact of demographic variables on elderly behavior remains a subject of academic debate. Consequently, this study incorporates commonly utilized gerontological demographic variables, such as gender, age, education, health, and income, as control variables. The theoretical model and associated hypotheses are depicted in Figure 1.

### 3.2 Hypothesis development

#### 3.2.1 Perceived usefulness and elderly m-banking engagement

Perceived usefulness reflects the assessment of a technology’s effectiveness in achieving personal goals (Yu et al., 2022). Research indicates a strong positive link between perceived usefulness of an information system and the likelihood of adoption, with users more inclined to adopt systems they find useful (Hu et al., 2024). In the context of this investigation, perceived usefulness encapsulates the anticipated benefits that older adults expect to derive from engaging with m-banking services. Such benefits include enhanced daily convenience, as m-banking empowers seniors to manage financial operations with ease, such as reviewing account balances and executing transactions, thereby augmenting the ease of managing daily financial affairs (Choudrie et al., 2020). Additionally, m-banking is perceived as bolstering financial security by offering a secure environment for transactions, enabling real-time account monitoring and fortifying the users’ financial confidence (Neves et al., 2024). The greater the perceived usefulness attributed to m-banking by older adults, the more inclined they are to utilize these services. Therefore, Hypothesis 1 is proposed:

*H1*: The perceived usefulness of m-banking among older adults is positively related to their m-banking engagement.

#### 3.2.2 Social influence triggers and perceived use

The elderly’s perceived usefulness of m-banking services is significantly affected by social interactions, including WOMs from trusted source (Zhang et al., 2023), peer tech engagement (Colosimo and Badia, 2021), and social norms (Ma et al., 2023). WOMs can prompt elderly individuals to internalize the perceived usefulness of technology by underscoring its advantages and alleviating any apprehensions (Zhang et al., 2023). This is especially relevant for those without extensive technological knowledge, who frequently depend on social endorsements to assess the applicability of m-banking services. Also, peer engagement acts as a significant catalyst in the social influence process known as identification, where individuals are influenced by observing and adopting the behaviors of others within their social networks (Yang et al., 2023). Observing peers adopting mobile banking (m-banking) can assist elderly individuals in overcoming their technological reservations and biases, leading them to perceive the technology as beneficial (Wong et al., 2022). Furthermore, it can encourage older adults to view the service as essential for maintaining social alignment and capital, thereby enhancing its perceived value (Burholt et al., 2023; Sauciuc and Persson, 2023), thereby increasing their perceived usefulness of m-banking services. Moreover, subjective norms, which are the perceived expectations of one’s social circle, significantly shape the elderly’s perspective on technology and prompt compliance with technological adoption (Zhang et al., 2023). For the elderly population, the expectations and opinions of friends and family, as well as societal views, hold particular importance (Ma et al., 2023). When seniors believe that their community expects them to use m-banking, they may anticipate positive social outcomes, further amplifying the service’s perceived use. Therefore, the following hypotheses are proposed:

*H2a*: The WOMs regarding m-banking received by older adults from their social relationships is positively associated with their perceived usefulness of m-banking.

*H2b*: Peer engagement is positively associated with older adults' perceived usefulness of m-banking.

*H2c*: Subjective norms are positively associated with older adults' perceived usefulness of m-banking.

#### 3.2.3 The moderating roles of digital literacy

Digital literacy plays a moderating role in the process by which individuals are influenced by social factors, as explained by the elaboration likelihood model (Jeyaraj et al., 2023). This model distinguishes between two pathways of information processing: the central route and the peripheral route, which differ in the amount of cognitive effort required (Arshad et al., 2024). The central route involves individuals forming judgments about products or services through in-depth analysis and evaluation of information. This process demands a high level of information search, processing skills, and willingness to engage in critical thinking, leading to a more comprehensive understanding (Zhang et al., 2024). On the other hand, the peripheral route relies on authoritative or credible information sources, simplifying the information processing effort and reducing cognitive exertion, making it more suitable for individuals with lower digital literacy (Chen et al., 2024). Therefore, older adults with lower digital literacy may lack the necessary skills and confidence to independently assess and understand new technologies, leading them to rely more on interpersonal information from their social networks (Zhang et al., 2023). These types of informational resources encompass suggestions from families and acquaintances (WOMs), prevalent practices among social circles (peer engagement), as well as perceived societal or communal expectations (subjective norms) from others. While information from peripheral routes is easily accessible and manageable, it may not be as comprehensive and in-depth as that obtained through the central route (Zhang et al., 2024). As the digital literacy of individuals increases, they are more likely to adopt the central route for decision-making (Jeyaraj et al., 2023). When evaluating and adopting emerging services such as m-banking, old adults with higher digital literacy tend to base their decisions on their own understanding of the technology, personal needs, and preferences, rather than solely on the opinions and suggestions of others. This autonomous decision-making process helps older adults to gain a deeper understanding and trust in new technologies, thereby enabling them to understand and use these technologies more independently (Choudrie et al., 2020). Therefore, the following hypotheses are proposed:

*H3a*: The digital literacy of older adults has a negative moderating effect on the positive relationship between WOMs and the perceived usefulness of m-banking services for older adults.

*H3b*: The digital literacy of older adults has a negative moderating effect on the positive relationship between peer engagement and the perceived usefulness of m-banking services for older adults.

*H3c*: The digital literacy of older adults has a negative moderating effect on the positive relationship between subjective norms and the perceived usefulness of m-banking services for older adults.

## 4 Data collection and scale development

### 4.1 Data collection procedure

Data was gathered through an online platform utilizing a disjunctive approach to prevent common method bias (Blau, 1999). The data collection was conducted in two phases, with the distribution of questionnaires facilitated by the Credamo platform,^1^ known for its system-generated random sample allocation and large participant pool, is utilized by numerous studies (Ma and Li, 2024; Zhang et al., 2024). In the first round, questionnaires were given to the elderly, including questions about social interaction, digital literacy-related variables, demographic information, contact details (WeChat or phone), and specific m-banking services they had heard of or used. In the second round, questionnaires containing questions about perceived usefulness, and m-banking services engagement were distributed to those who had clearly answered all questions in the first round. The interval between the two surveys was 24–48 h. From February to May 2023, a total of 500 questionnaires were distributed, with 414 valid questionnaires collected in the first round and 249 in the second round. Questionnaires with identical answers to all questions or more than 15% unanswered were considered invalid. Additionally, due to the author’s involvement in numerous WeChat groups for elderly services, questionnaires were also distributed to the elderly in these groups to extend the distribution. A total of 1,000 questionnaires were distributed online, with 298 valid questionnaires returned. When collecting data online, the order of questions was altered, with questions about intentions to use m-banking services placed at the beginning of the questionnaire, and reverse questions added at the end to test the validity of the questionnaire. Questionnaires with identical answers to all questions, more than 15% unanswered, or with reverse question answers that did not correspond were considered invalid, as were questionnaires from individuals under 50 years old. The overall valid response rate for questionnaires was 28.33% with 425 valid answers. Harman’s single-factor test (Yang et al., 2023) was used to verify the presence of common method bias in the questionnaire data. The test results showed several distinct factors, with the first factor explaining less than the 40% threshold of variance. Therefore, there was no severe common method bias. The demographic characteristics of the sample are presented in Table 2.

**Table 2:** Demographic distribution of the survey respondents.

Demographic information		Frequency	Percentage(%)
Gender	Male	225	52.94
	Women	200	47.06
Age	50–59	130	30.59
	60–69	184	43.29
	70–79	83	19.53
	≥80	28	6.59
Education	Junior high school and below	20	4.76
	High school	210	49.37
	University (Undergraduate/Junior College)	178	41.85
	Graduate student or above	17	4.02
Health	Healthy	35	8.24
	Average	242	56.94
	Have an illness	148	34.82
	Need for long-term care	0	0
Incomes	Less than ¥1,500/month	0	0
	1,500–3,000 RMB/month	42	9.88
	3,000–5,000 RMB /month	305	71.76
	5,000–8,000 RMB/month	75	17.65
	More than 8,000 RMB/month	3	0.71

### 4.2 Scale design

The measurement indicators for the various variables in the scale were derived from existing scales used in prior research, ensuring their reliability and validity through a two-stage process of content and face validity testing. In the first stage, three academic experts in the fields of e-commerce and elderly care services were invited to assess the face and content validity of the measurement indicators for each variable. The percentage of absolute agreement was used to measure the internal consistency among raters (Graham et al., 2012). The study found that the absolute agreement rates for all variable measurement indicators ranged from 90 to 100%, indicating that the experts unanimously agreed that the majority of the measurement items could represent the corresponding variables. In the second stage, a pretest was conducted with 20 elderly individuals to evaluate the face validity of the questionnaire items, assess the logical consistency and understandability among the indicators, and refine the specific wording of each measurement item. After revisions, all elderly participants agreed that the questionnaire accurately reflected the characteristics of the platforms they had heard of or joined, and that the wording was easy to understand.

Table 3 presents the final version of the scale. The three indicators of WOMs are based on Lin et al. (2019) scale, the three indicators of peer engagement are adapted from Strader et al. (2007), and the four indicators of subjective norms are derived from Niehaves and Plattfaut (2014). The four indicators of digital literacy are developed based on Noh (2017) study. The six indicators of perceived usefulness are derived from Wong et al. (2022) study, and the three indicators of perceived ease of use are adapted from Wong et al. (2022) scale. Elderly m-banking engagement is composed of three indicators, adapted from the scale developed by Guo et al. (2016). All questionnaire items are evaluated using a five-point Likert scale, ranging from 1 to 5 (“strongly disagree” to “strongly agree”).

**Table 3:** Scales.

Variables	Items	References
WOMs	Many people around you recommend this m-banking. Your friends recommend this m-banking. Your friends and family encourage you to use this m-banking.	Lin et al. (2019)
Peer engagement	Many people around you use this m-banking. Many of your friends use this m-banking. Your family members and relatives often use this m-banking.	Strader et al. (2007)
Subjective norms	Your friends and family think you should use this m-banking with them. Your friends and family persuade you to use this m-banking to interact with others. Many people around you think you should use this m-banking with them. Many people around you persuade you to use this m-banking. Many people around you have encouraged you to use this platform.	Neves et al. (2024)
Digital literacy	How would you rate your ability to use the Internet or social networking tools to communicate with others? How often do you need the help of others when doing network operations or using software? How would you rate your ability to use Internet tools to search for information? Do you have the ability to select, analyze, and organize digital information from a variety of sources and convert it into necessary knowledge?	Sakariassen and Ytre-Arne (2024)
Perceived usefulness	This m-banking facilitates access to needed information. This m-banking facilitates your access to financial information. This m-banking is convenient for transferring and receiving funds This m-banking service allows for easy online payments. This m-banking service can provide you with financial assistance. This m-banking service is beneficial for planning and managing your financial activities.	Wong et al. (2022)
Perceived ease of use	Using this m-banking is easy. It is not considered difficult to conduct financial activities using this m-banking. You can easily figure out the features of this m-banking.	Wong et al. (2022)
Elderly M-banking engagement	You’ll be exploring m-banking features in the near future You will consistently using various services of m-banking.You will be willing to interact with others though m-banking.	Saif et al. (2024)

### 4.3 Results

This study employs Partial Least Squares Structural Equation Modeling (PLS-SEM) as the research method. There are three reasons for choosing PLS-SEM. First, PLS-SEM is suitable for analyzing complex models involving multiple variables (Hair et al., 2019). Second, PLS-SEM can be used to analyze research that integrates various theories (Leguina, 2015). Third, PLS-SEM is appropriate for data that is not normally distributed. This technique can yield robust results even when the data is highly skewed (Ringle et al., 2014). The study adopts a two-step approach consisting of measurement model testing and structural model assessment to test the proposed hypotheses.

#### 4.3.1 Measurement model

Reliability is assessed using Cronbach’s alpha. As shown in Table 4, all variables have Cronbach’s alpha values greater than the threshold of 0.7, indicating good reliability. Convergent validity is evaluated based on three criteria: factor loadings, average variance extracted (AVE), and composite reliability (CR). The threshold values for factor loadings, AVE, and CR are 0.7, 0.5, and 0.7, respectively (Hair et al., 2019). As demonstrated in Table 4, all convergent validity criteria exceed the thresholds. Discriminant validity is assessed using the Fornell-Larcker criterion and the heterotrait-monotrait ratio (HTMT). As shown in Table 5, the square root of AVE for all variables is greater than the inter-variable correlation coefficients. As indicated in Table 6, all HTMT values are below the threshold of 0.9, suggesting good discriminant validity for all variables.

**Table 4:** Construct validity and reliability.

Variable	Factor loading		α	CR	AVE
WOMs	WOM1	0.715	0.792	0.865	0.616
	WOM2	0.773			
	WOM3	0.805			
	WOM4	0.842			
Peer engagement	PRE1	0.932	0.838	0.903	0.758
	PRE2	0.794			
	PRE3	0.880			
Subjective norms	SN1	0.832	0.851	0.900	0.692
	SN2	0.850			
	SN3	0.779			
	SN4	0.865			
Digital literacy	DIG1	0.965	0.969	0.977	0.914
	DIG2	0.959			
	DIG3	0.948			
	DIG4	0.953			
Perceived usefulness	PU1	0.756	0.837	0.916	0.644
	PU2	0.825			
	PU3	0.801			
	PU4	0.824			
	PU5	0.793			
	PU6	0.817			
Perceived ease of use	PEOU1	0.884	0.889	0.902	0.754
	PEOU2	0.878			
	PEOU3	0.843			
Elderly m-banking Engagement	ENG1	0.862	0.713	0.838	0.634
	ENG 2	0.737			
	ENG 3	0.786			

**Table 5:** Matrix of correlation coefficients of variables and square root of AVE.

Variable	1	2	3	4	5	6	7	8	9	10	11	12
1. Gender												
2. Age	0.043^NS^											
3 Education	-0.022^NS^	−0.212^***^										
4. Health	−0.038^NS^	−0.099^**^	0.074^NS^									
5. Income	−0.020^NS^	0.061^NS^	0.121^NS^	0.299								
6. WOMs	0.008^NS^	−0.065 ^NS^	0.021^NS^	−0.032^NS^	−0.080^NS^	**0.785**						
7. Peer engagement	0.025^NS^	−0.019 ^NS^	0.049^NS^	−0.039^NS^	−0.073^NS^	0.576^***^	**0.871**					
8. Subjective norms	−0.107^*^	0.093^NS^	−0.024^NS^	0.001^NS^	0.090^NS^	0.552^***^	0.465^***^	**0.832**				
9. Digital literacy	0.049^NS^	−0.116^*^	0.131^**^	0.036^NS^	−0.064^NS^	−0.007^NS^	0.031^NS^	−0.015 ^NS^	**0.634**			
10. Perceived usefulness	0.034^NS^	0.043^NS^	−0.040^NS^	0.013^NS^	0.004^NS^	0.506^**^	0.474^***^	0.304^***^	−0.034^NS^	**0.803**		
11. Perceived ease of use	−0.076^NS^	0.105^*^	−0.089^NS^	0.090^NS^	0.102^*^	0.331^***^	0.381^***^	0.206^***^	0.627^***^	0.374^***^	**0.868**	
12. Elderly M-banking Engagement	0.018 ^NS^	−0.014 ^NS^	−0.037^NS^	0.021^NS^	−0.035^NS^	0.603^***^	0.556^***^	0.408^***^	0.209^***^	0.455^***^	0.527^***^	**0.930**
Average	0.471	63.424	2.435	3.734	3.092	3.912	3.666	3.872	2.574	3.779	3.449	3.681
Standard deviation	0.499	9.511	0.644	0.600	0.543	0.597	0.495	0.631	1.394	0.477	0.471	0.511

Bold numbers on the diagonal are the square root of the variable’s AVE. Sex is coded as 1 for female and 0 for male; education is coded as 1 for middle school or less, 2 for high school, 3 for bachelor’s degree, and 4 for master’s degree or higher; health is coded as 1 for needing long term care, 2 for having a medical condition, 3 for general, and 4 for being healthy; and income is coded as 1 for having a monthly income of less than ¥1,500, 2 for having a monthly income between ¥1,500 and ¥3,000, 3 indicates monthly income between ¥3,000 and ¥5,000, 4 indicates monthly income between ¥5,000 and ¥8,000, and 5 indicates monthly income of ¥8,000 or more. ***p = 0.001, **p = 0.01, *p = 0.05, NS, not significant (based on two-tailed student t(4999) distribution).

**Table 6:** Heterogeneous-to-monomers ratios (HTMT).

Variable	1	2	3	4	5	6	7	8	9	10	11	12
1. Gender												
2. Age	0.043											
3. Education	0.022	0.212										
4. Health	0.038	0.105	0.074									
5. Income	0.020	0.067	0.121	0.299								
6. WOMs	0.028	0.073	0.023	0.038	0.093							
7. Peer engagement	0.038	0.035	0.052	0.043	0.082	0.709						
8. Subjective norms	0.053	0.126	0.043	0.039	0.069	0.672	0.552					
9. Digital literacy	0.058	0.094	0.144	0.013	0.091	0.049	0.036	0.035				
10. Perceived usefulness	0.035	0.105	0.048	0.028	0.042	0.598	0.547	0.349	0.146			
11. Perceived ease of use	0.082	0.114	0.097	0.034	0.111	0.408	0.461	0.244	0.693	0.432		
12. Elderly M-banking Engagement	0.019	0.015	0.040	0.023	0.038	0.734	0.662	0.481	0.230	0.523	0.627	

Before conducting hypothesis testing, multicollinearity of variables was analyzed using the variance inflation factor (VIF) for the variables. It is widely accepted in academia that a VIF value less than 10 indicates no severe multicollinearity. As shown in Tables 7, 8, the range of VIF values for all variables is less than 10, indicating that there is no severe multicollinearity in the model (Leguina, 2015).

**Table 7:** Measurement model multicollinearity test.

Variables		VIF
WOMs	WOM1	1.385
	WOM2	1.602
	WOM3	1.737
	WOM4	1.852
Peer engagement	PRE1	3.332
	PRE2	1.617
	PRE3	2.702
Subjective norms	SN1	2.831
	SN2	2.939
	SN3	2.039
	SN4	2.572
Digital literacy	DIG1	4.417
	DIG2	4.276
	DIG3	4.794
	DIG4	4.523
Perceived usefulness	PU1	1.725
	PU2	2.223
	PU3	2.153
	PU4	2.176
	PU5	2.036
	PU6	2.278
Perceived ease of use	PEOU1	2.153
	PEOU2	2.016
	PEOU3	1.799
Elderly M-banking engagement	ENG1	2.150
	ENG 2	2.150
	ENG3	1.899

**Table 8:** Structural model multicollinearity test.

Variable	1	2	3	4	5	6	7	8	9	10	11	12
1. Gender												1.015
2. Age												1.091
3. Education												1.080
4. Health												1.126
5. Income												1.144
6. WOMs										1.865		
7. Peer engagement										1.736		
8. Subjective norms										1.515		
9. Digital literacy										1.895	1.000	
10. Perceived usefulness												1.170
11. Perceived ease of use										2.305		1.199
12. Elderly m-banking engagement												

#### 4.3.2 Structural model

##### 4.3.2.1 Direct effects

The path coefficients (β) in terms of their signs and significance were used as indicators to test whether the hypotheses were supported (Ali et al., 2018). The signs of the path coefficients should be consistent with the hypotheses and be significant at a 95% confidence level. As indicated in Table 9, the perceived usefulness of m-banking services by older adults is significantly positively correlated with their m-banking engagement (*β* = 0.294, *p* < 0.001), supporting H1. WOMs is significantly positively correlated with the perceived usefulness of m-banking services by older adults (*β* = 0.288, *p* < 0.001), supporting H2a. Peer engagement is significantly positively correlated with the perceived usefulness of m-banking services by older adults (*β* = 0.219, *p* < 0.001), supporting H2b. The relationship between subjective norms and the perceived usefulness of m-banking services by older adults is not significant (*β* = −0.033, *p* > 0.05), hence H2c is not supported.

**Table 9:** Hypothesis testing.

Paths	β	*t*-values	Conclusion
Gender→Elderly m-banking engagement	0.042	1.119 ^NS^	
Age→Elderly m-banking engagement	−0.064	1.726 ^NS^	
Education→Elderly m-banking engagement	0.008	1.139 ^NS^	
Health→Elderly m-banking engagement	0.002	0.056^NS^	
Income→Elderly m-banking engagement	0.069	1.649 ^NS^	
Perceived ease of use→Elderly m-banking engagement	0.433	10.96***	
Perceived ease of use→Perceived usefulness	0.292	5.204***	
H1 Perceived usefulness→Elderly m-banking engagement	0.294	6.706***	Support
H2a WOMs→Perceived usefulness	0.288	5.030***	Support
H2b Peer engagement→Perceived usefulness	0.219	3.546***	Support
H2c Subjective norms→Perceived usefulness	−0.033	0.70 ^NS^	Not support
H3a WOMs * digital literacy→perceived usefulness	−0.204	3.045***	Support
H3b Peer engagement* Digital literacy→Perceived usefulness	−0.167	2.764***	Support
H3c Subjective norms* Digital literacy→Perceived usefulness	−0.067	1.346 ^NS^	Not support

Bold numbers on the diagonal are the square root of the variable’s AVE. Gender is coded as 1 for female and 0 for male; education is coded as 1 for middle school or less, 2 for high school, 3 for bachelor’s degree, and 4 for master’s degree or higher; health is coded as 1 for needing long term care, 2 for having a medical condition, 3 for general, and 4 for being healthy; and income is coded as 1 for having a monthly income of less than ¥1,500, 2 for having a monthly income between ¥1,500 and ¥3,000, 3 indicates monthly income between ¥3,000 and ¥5,000, 4 indicates monthly income between ¥5,000 and ¥8,000, and 5 indicates monthly income of ¥8,000 or more. ***p = 0.001, **p = 0.01, *p = 0.05, NS, not significant (based on two-tailed student t(4999) distribution).

##### 4.3.2.2 Moderation effect

As shown in Table 9, the interaction term between WOMs and the digital literacy of older adults is significantly negatively correlated with the perceived usefulness of m-banking services by older adults, thus supporting H3a (*β* = −0.204, *p* < 0.001). The interaction term between peer engagement and the digital literacy of older adults is also significantly negatively correlated with the perceived usefulness of m-banking services by older adults (*β* = −0.167, *p* < 0.001), thus supporting H3b. The interaction term between subjective norms and the digital literacy of older adults is significantly negatively correlated with the perceived usefulness of m-banking services by older adults (*β* = −0.067, *p* > 0.05), which does not support H3c.

**Figure 2:**
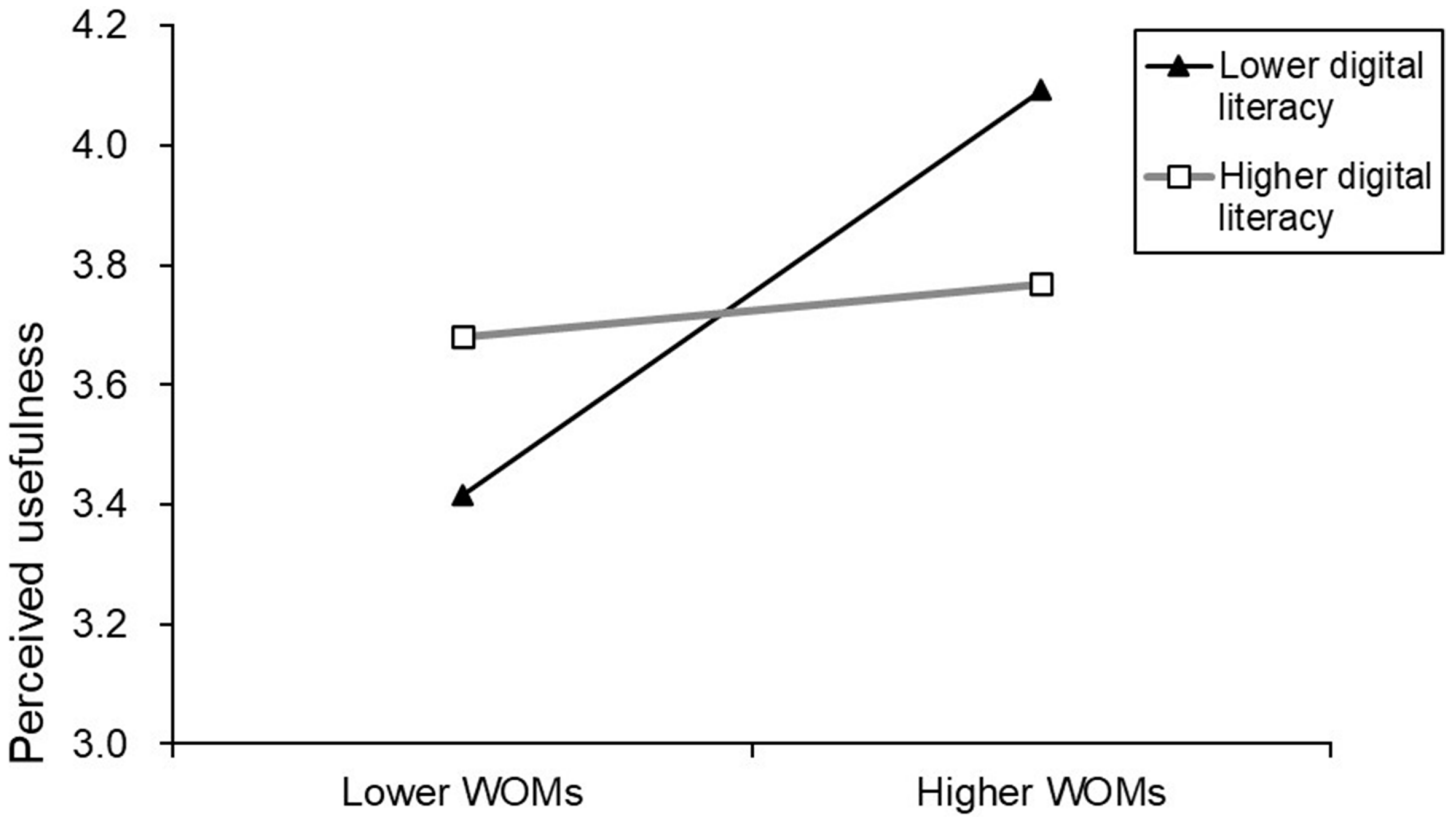
Simple slope analysis plot of digital literacy, WOMs and perceived usefulness.

**Figure 3:**
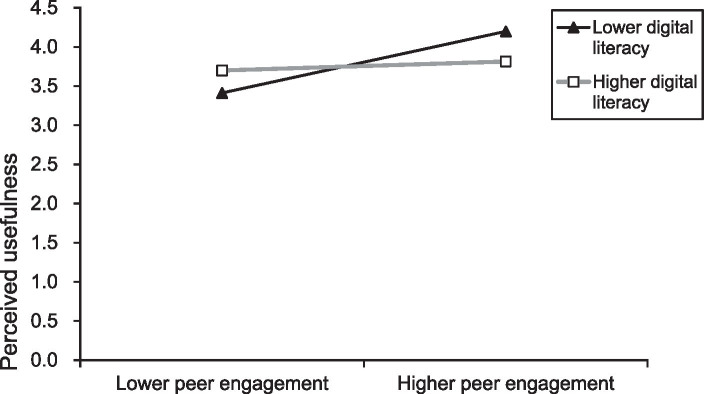
Simple slope analysis plot of digital literacy, peer engagement, and perceived usefulness.

The study further employed simple slope analysis (Wang et al., 2022) to delve deeper into the moderating effects of digital literacy on the relationship between WOMs and the perceived usefulness of m-banking services by older adults, as well as the moderating effects on the relationship between peer engagement and the perceived usefulness of m-banking services by older adults. The results indicate that under high levels of digital literacy (one standard deviation above the mean), the positive correlation between WOMs and perceived usefulness is lower than under low levels of digital literacy (one standard deviation below the mean; simple slope = 0.089, *p* < 0.001; simple slope = 0.677, *p* < 0.001). Similarly, under high levels of digital literacy, the positive correlation between peer engagement and perceived usefulness is lower than under low levels of digital literacy (simple slope = 0.116, *p* < 0.001; simple slope = 0.796, *p* < 0.001). Figures 2, 3 present the corresponding simple slope analysis graphs.

##### 4.3.2.3 Model fit

The study further assesses the robustness of the model. Stone-Geisser’s Q2 is utilized to examine the model’s predictive relevance. As indicated in Table 10, the Stone-Geisser’s Q2 values for perceived usefulness, perceived ease of use, and intention to use m-banking services are 0.324, 0.291, and 0.303, respectively, all greater than the threshold of 0, suggesting that the model has a certain level of predictive relevance. The goodness of fit (GoF) value of the model is 0.722, which indicates a substantial model fit. The explained variance (R^2^) of the endogenous constructs is used to evaluate the extent to which the dependent variables are accounted for by the independent variables. The R^2^ values for perceived usefulness, perceived ease of use, and intention to use m-banking services are 0.506, 0.392, and 0.356, respectively, all exceeding the threshold of 0.1. In summary, the structural model of this study is robust.

**Table 10:** Model fit.

	SSO	SSE	Q^2^ (=1-SSE/SSO)
1. Gender	425.000	425.000	
2. Age	425.000	425.000	
3. Education	425.000	425.000	
4. Health	425.000	425.000	
5. Income	425.000	425.000	
6. WOMs	1700.000	1700.000	
7. Peer engagement	1275.000	1275.000	
8. Subjective norms	1700.000	1700.000	
9. Digital literacy	1697.000	1697.000	
10. Perceived usefulness	2550.000	1724.822	0.324
11. Perceived ease of use	1275.000	903.581	0.291
12. Elderly m-banking engagement	850.000	592.668	0.303

## 5 Discussion and implications

### 5.1 Conclusion

This study aims to explore the mechanisms by which older adults engage with m-banking services under the influence of social interaction. The research findings indicate that WOMs and peer engagement, as forms of social interaction, positively influence the older adults’ perceived usefulness of m-banking services, which affects their m-banking engagement. Digital literacy negatively moderates the positive effects of WOMs and peer engagement on the perceived usefulness of m-banking services for older adults. These results suggest that the model proposed in this paper can explain the process by which older adults with different levels of digital literacy engage with m-banking services under the influence of social interaction.

The research reveals the positive influence of WOMs and peer engagement on the perceived usefulness of m-banking services for older adults, while the impact of subjective norms on perceived usefulness is not proven. This is different from studies focusing on young people, such as those by Pescher et al. (2014), Chica and Rand (2017), which often consider subjective norms as the sole variable representing social influence. For most older adults, m-banking engagement is challenging, and they are unlikely to incur high learning costs to accommodate others’ wishes. Nonetheless, WOMs can enlighten older individuals about the benefits they can accrue that justify the effort required to learn about them. Additionally, the involvement of their peers can vividly demonstrate the social backing they are likely to receive through the utilization of m-banking services, thereby helping them to appreciate the practicality of such services.

The study uncovers the moderating role of digital literacy in older adults’ decision-making behavior. Higher digital literacy negatively moderates the positive correlation between social interaction (WOMs, peer engagement) and perceived usefulness of m-banking services by older adults, as those with higher digital literacy have broader channels for obtaining information, stronger independent thinking, and more independent judgments regarding the usefulness of the services.

### 5.2 Theoretical contributions

This research investigates the pathways and influencing factors through which platform users lead elderly m-banking engagement. Previous studies on user participation in social platforms have primarily focused on young people (Malarvizhi et al., 2022), examining the impact of social connections and interactions among existing users on their consequent behavior (Hayat et al., 2022). Older adults are expected to become potential customers of m-banking (Wang et al., 2022), as these individuals often show a strong desire for support and guidance from their social circles, particularly the assistance from friends and family, to facilitate their engagement with m-banking services (Ma et al., 2023). Therefore, this study shifts the research perspective to older adults, investigating the mechanisms by which platform users lead potential users (i.e., older adults offline) to engage with m-banking services online, enriching the research on m-banking engagement.

Guided by integrating social influence theory, this study identifies the effectiveness of two social influence processes (based on internalization and identification) in explaining the perceived usefulness of m-banking services by older adults. While classic models and research on the antecedents of individual online activities have focused on the social influence process based on compliance (Shen et al., 2023; Cui et al., 2024; Tian et al., 2024) which consider subjective norms as a key external variable affecting individuals’ acceptance of information technology. In contrast, the social influence processes based on identification and internalization have been less studied, yet they are common in the elderly population. Platform users can engage in various forms of social interaction with older adults offline with whom they have established social connections in real life, such as through WOMs to familiarize offline older adults with online services (social influence based on internalization); the behavior of online users themselves in engaging with online services also affects older adults’ cognition of m-banking services and subsequent behavior (social influence based on identification). Moreover, most older adults require a higher threshold to accept new information technology, making subjective norms less likely to drive older adults to adopt new information technology (compliance-based influence mechanism). This study reveals the importance of identification and internalization-based social influences for older adults’ digital engagement, uncovering the social influence processes in older people’s digital engagement and enriching the research on information technology acceptance under social influence.

The study finds that digital literacy has an important moderating impact on older adults’ engagement. Digital literacy negatively moderates the positive correlation between social interaction and perceived usefulness, which is an essential psychological mechanism for older adults to participate in online services. Previous research has mainly focused on the direct impact of digital literacy on older adults’ participation in online activities or acceptance of information technology, emphasizing the negative effects of digital literacy deficiencies (Wong et al., 2022; Maceviciute et al., 2023). The potential moderating role of digital literacy between older adults’ digital engagement and their driving factors has been little studied. This study’s finding of the negative moderating effect of digital literacy on the social influence of platform users clarifies the boundary conditions for platform users to lead older adults offline to participate in online services, revealing the profile of the elderly population targeted by this path. Furthermore, this study finds that the social interaction of platform users has a better effect on older adults with lower levels of digital literacy. This finding challenges the existing perception that digital literacy deficiencies necessarily hinder older adults’ digital engagement, providing a new analytical dimension for researching the relationship between digital literacy and older adults’ online participation behavior.

### 5.3 Practical implications

The study finds that older adults’ perceptions of the usefulness of m-banking services significantly influence their engagement. Platform leaders need to carefully design platform features that meet the value pursuits of older adults, providing them with sufficient information and social support, but without overly complicating the platform’s operations. This requires platform leaders to conduct detailed market research in advance and continuously track user data to improve platform design, creating a practical and user-friendly financial service platform for older adults.

The study reveals the impact of WOMs and peer engagement on older adults’ perceived usefulness of m-banking services, while the relationship between subjective norms and perceived usefulness is not significant. Therefore, when platform users guide older adults around them to participate in m-banking services online, they should not simply encourage and advocate. Instead, by using some social marketing strategies, mobilize existing users to vividly explain the advantages of mobile banking to the elderly, and demonstrate their own successful practices in using mobile banking to them. Platform leaders and service providers can also design activities that involve both potential and actual users of the platform, making older users of the platform more willing to share information about the platform with surrounding older adults and demonstrate the specific ways to participate in the services, making it easier for older adults to recognize the platform’s value.

The study uncovers the moderating role of digital literacy in older adults’ decision-making behavior. Although older adults with lower digital literacy may perceive the learning cost of using the platform as too high, they are more susceptible to the influence of social factors. Therefore, platform leaders and relevant personnel can tailor their promotional strategies based on the digital literacy levels of their target audience. For instance, to bridge the digital divide among older adult groups, the platform needs to enhance the guidance from social factors and intensify its social marketing efforts specially for the elderly with lower digital literacy. For example, the platform can be designed to be simple, and interactions with platform users can encourage them to continuously spread the platform to surrounding older adults. Encouraging adoption by highlighting the experiences of peers who have successfully integrated the platform into their lives. For older adults with higher digital literacy, more diverse promotional methods can be adopted, such as mobilizing offline service providers of the platform to promote it to older adults and using digital media and other information channels to provide more detailed and comprehensive introductions to the platform’s functions, thereby enhancing the platform’s attractiveness to older adults.

### 5.4 Limitations and future work

Although this study has many valuable findings and implications, it remains preliminary and includes several limitations. First, this study used a survey as the main research method. Other methods, such as experiments, can be used to cross-validate the conclusions. Second, the questionnaires were completed by the same person during the survey process due to the purpose and nature of the study. Although we have done some control and post-testing for common method variance, it cannot be eliminated. Further, research can collect data from multiple sources. Third, this study may not include all the important variables. Further research can consider other variables, such as organizational performance indicators, to provide managers with more practical implications. Fourth, surveys inherently face challenges in achieving perfect randomness, future studies should aim to forge partnerships with a broader array of research institutions, data collection agencies, and governmental entities. This collaboration can enhance the scientific rigor and scale of sampling methodologies, leading to more robust and representative research outcomes. Furthermore, despite the incorporation of control variables—a standard approach in the literature to address endogeneity in structural equation modeling—achieving complete mitigation of endogeneity remains elusive. Further research should endeavor to incorporate experimental methodologies to fortify the robustness of our conclusions.

## Data availability statement

The raw data supporting the conclusions of this article will be made available by the authors, without undue reservation.

## Ethics statement

The studies involving humans were approved by Ethics Commission of the Faculty of Criminal Justice and Security, Zhejiang University of Finance & Economics. The studies were conducted in accordance with the local legislation and institutional requirements. The participants provided their written informed consent to participate in this study. Written informed consent was obtained from the individual(s) for the publication of any potentially identifiable images or data included in this article.

## Author contributions

YM: Writing – original draft, Writing – review & editing.

## Footnotes

^1^ https://www.credamo.com/
